# Autosomal Recessive Rod–Cone Dystrophy with Mild Extra-Ocular Manifestations Due to a Splice-Affecting Variant in *BBS9*

**DOI:** 10.3390/cimb46030163

**Published:** 2024-03-18

**Authors:** Iris Deitch, Sofia Itskov, Daan Panneman, Aasem Abu Shtaya, Tal Saban, Yael Goldberg, Miriam Ehrenberg, Frans P. M. Cremers, Susanne Roosing, Tamar Ben-Yosef

**Affiliations:** 1 Rabin Medical Center, Department of Ophthalmology, Petach Tikva 4941492, Israel; iris.deitch@gmail.com (I.D.); talm1@clalit.org.il (T.S.); 2 Faculty of Medicine, Tel Aviv University, Tel Aviv 6997801, Israel; yaelgo43@clalit.org.il (Y.G.); mirieh1@clalit.org.il (M.E.); 3 Ruth & Bruce Rappaport Faculty of Medicine, Technion-Israel Institute of Technology, Haifa 3109601, Israel; sofia.itskov@campus.technion.ac.il; 4 Department of Human Genetics, Radboud University Medical Center, 6525 GA Nijmegen, The Netherlands; daan.panneman@radboudumc.nl (D.P.); frans.cremers@radboudumc.nl (F.P.M.C.); susanne.roosing@radboudumc.nl (S.R.); 5 Raphael Recanati Genetic Institute, Rabin Medical Center, Beilinson Hospital, Petach Tikva 4941492, Israel; asemab@clalit.org.il; 6 Unit of Gastroenterology, Carmel Medical Center, Haifa 3436212, Israel; 7 Schneider Children’s Medical Center of Israel, Department of Ophthalmology, Petach Tikva 4920235, Israel

**Keywords:** retina, retinitis pigmentosa, rod–cone dystrophy, *BBS9*, Bardet–Biedl syndrome

## Abstract

Bardet–Biedl syndrome (BBS), one of the most common forms of syndromic inherited retinal diseases (IRDs), is characterized by the combination of retinal degeneration with additional extra-ocular manifestations, including obesity, intellectual disability, kidney disease, polydactyly and other skeletal abnormalities. We observed an Israeli patient with autosomal recessive apparently non-syndromic rod–cone dystrophy (RCD). Extra-ocular findings were limited to epilepsy and dental problems. Genetic analysis with a single molecule molecular inversion probes-based panel that targets the exons and splice sites of 113 genes associated with retinitis pigmentosa and Leber congenital amaurosis revealed a homozygous rare missense variant in the *BBS9* gene (c.263C>T;p.(Ser88Leu)). This variant, which affects a highly conserved amino acid, is also located in the last base of Exon 3, and predicted to be splice-altering. An in vitro minigene splice assay demonstrated that this variant leads to the partial aberrant splicing of Exon 3. Therefore, we suggest that this variant is likely hypomorphic. This is in agreement with the relatively mild phenotype observed in the patient. Hence, the findings in our study expand the phenotypic spectrum associated with *BBS9* variants and indicate that variants in this gene should be considered not only in BBS patients but also in individuals with non-syndromic IRD or IRD with very mild extra-ocular manifestations.

## 1. Introduction

Inherited retinal diseases (IRDs) are a clinically heterogeneous group of disorders, which cause visual loss due to improper development, improper function or premature death of the retinal photoreceptors [1]. The most common form of IRD is retinitis pigmentosa (RP), also known as rod–cone dystrophy (RCD) [2]. IRDs can manifest as an isolated phenotype or as a clinical feature of a syndrome. In the majority of IRD cases, the disease is confined to the eye, resulting in a non-syndromic manifestation. Nevertheless, over 70 forms of syndromic IRD, in which other organs are involved, have been described [3].

One of the most common forms of syndromic IRD is Bardet–Biedl syndrome (BBS), an autosomal recessive condition with a prevalence of approximately 1/125,000 [4]. BBS is defined as a ciliopathy, i.e., a disease caused by variants in genes encoding proteins which are associated with the structure and/or function of primary cilia [5]. Consequently, despite genetic heterogeneity, individuals with BBS exhibit common clinical features, leading to the classification of the syndrome as a distinct entity. Specifically, BBS is characterized by a variable combination of symptoms, including retinal dystrophy, macular atrophy, postaxial polydactyly (alongside other skeletal abnormalities), hypogonadism, renal disease, intellectual disability and truncal obesity. Additional minor systemic manifestations may include neurologic, olfactory, dental, cardiovascular, gastrointestinal, endocrine and metabolic abnormalities [4].

At least 26 BBS causative genes have been reported to date (OMIM, https://www.omim.org/, accessed on 13 March 2024) [4]. Most of these genes can be broken into two groups: (a) Genes encoding subunits of a protein complex, the BBSome, which is integral in ciliary as well as intracellular trafficking [6,7]. In the retina, the BBSome is required for photoreceptor outer segment formation and maintenance [8], as well as for retinal synaptogenesis [9]. (b) Genes encoding chaperonin-like proteins, which are a part of the rapidly evolving, vertebrate specific branch of the type II chaperonin superfamily [10].

The *BBS9* gene (also known as parathyroid hormone responsive B1, *PTHB1*) was first identified in 2005, by means of homozygosity mapping of small, consanguineous BBS pedigrees, followed by comparative genomic analysis and gene expression studies of a BBS-knockout mouse model [11]. The BBS9 protein was shown to be a part of the BBSome complex [6], and its knock-down led to cilia defects in both zebrafish and mammalian cells [12]. To date, over 90 pathogenic *BBS9* variants have been reported worldwide (the Leiden Open Variation Database (LOVD); http://www.lovd.nl, accessed on 13 March 2024), and *BBS9* mutations were shown to account for 4–16% of BBS cases in various populations [13,14,15,16,17]. The vast majority of reported *BBS9* pathogenic variants were identified in patients with classical BBS. An exception is a family reported by Abu-Safieh et al., in which three siblings were homozygous for a deletion of *BBS9* Exon 6, which is expected to cause an in-frame deletion of 87 amino acids (p.(Gly148_Val234del)). Interestingly, the index patient had all the primary features of BBS, while her two sisters had non-syndromic RP [18]. Of note, variants in at least six BBS-causative genes have also been associated with non-syndromic RP/IRD. These include *BBS1* [19], *BBS2* [20], *ARL6* [21,22], *TTC8* [23,24], *IFT172* [25] and *CFAP418* (*C8orf37*) [26,27,28]. Here, we report a patient with RCD and very mild extra-ocular manifestations, due to a homozygous hypomorphic *BBS9* variant.

## 2. Case Presentation

**Figure 1:**
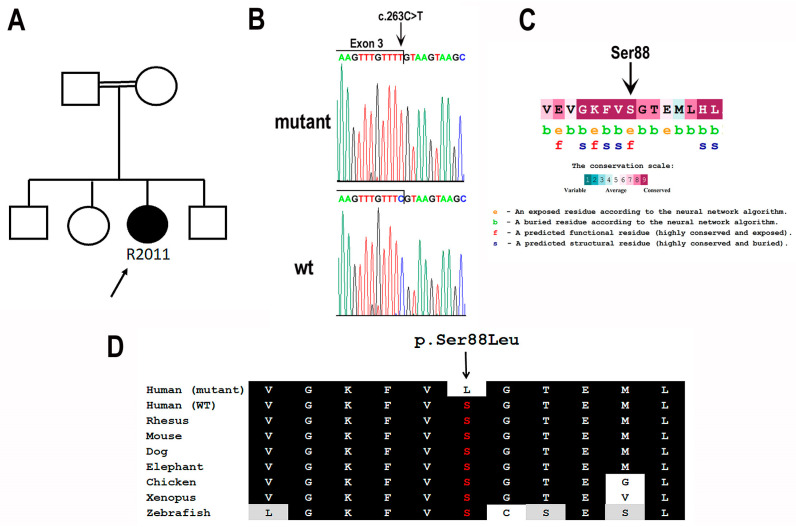
Identification of the *BBS9* c.263C>T;p.(Ser88Leu) variant. (**A**) Pedigree of Patient R2011. The black-filled symbol represents an affected individual, whereas clear symbols represent unaffected individuals. A double line represents a consanguineous marriage. (**B**) Mutant and wild-type (wt) nucleotide sequence traces of the boundary between *BBS9* Exon and Intron 3. The c.263C>T variant (marked by an arrow) affects the last base of Exon 3, which is part of the conserved donor splice-site. (**C**) Evolutionary conservation of BBS9 serine 88. The analysis was performed with The ConSurf Server [29] based on 272 BBS9 orthologues. The background color of each amino acid indicates its conservation score (see scale). Serine at position 88 has the highest conservation score, e, exposed amino acid; b, buried amino acid; f, functionally important amino acid (conserved and exposed); s, structurally important amino acid (conserved and buried). (**D**) Multiple sequence alignment of the region spanning the Ser88 amino acid of the BBS9 protein (highlighted in red and marked by an arrow) in various organisms. Conserved amino acids are indicated by a black background. Similar amino acids are indicated by a gray background. Also shown is the predicted sequence of the mutant protein harboring the p.Ser88Leu variant (first line).

**Figure 2:**
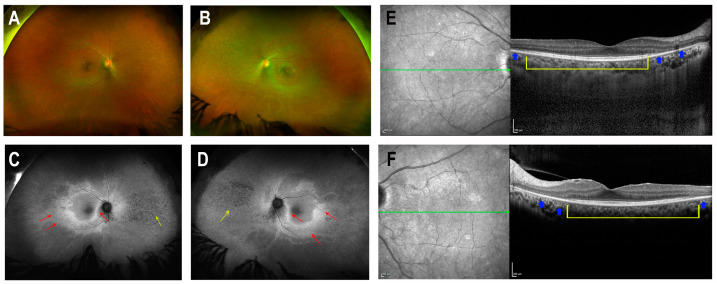
Clinical findings in Patient R2011. (**A**,**B**) Color fundus photographs (OD/OS), showing arterial attenuation, diffuse peripheral nasal depigmentation and inferior macular atrophy. (**C**,**D**) Fundus autofluorescence (OD/OS), revealing a bilateral symmetric pattern of the perifoveal hyperautofluorescent ring (red arrows) and midperipheral nasal hypoautofluorescent specks (yellow arrows). (**E**,**F**) Optical coherence tomography (OCT) of the macula (OD/OS), demonstrating preserved fovea (yellow brackets) with perifoveal ellipsoid zone loss (blue arrows). The green arrows on the left images indicate the location of the cross-sections shown on the right.

**Figure 3:**
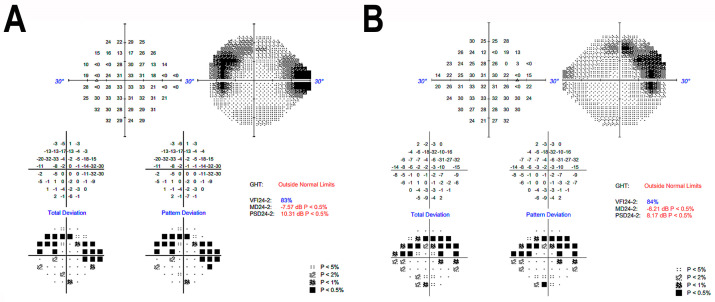
Humphrey visual field 24-2 of left eye (**A**) and right eye (**B**) eye, revealing mild symmetric constriction, characterized by bilateral upper arcuate scotoma, corresponding to the anatomic disruption in the macula.

A female patient (Patient R2011) from a consanguineous Israeli family of Iraqi Jewish descent (parents are first cousins) was ascertained by the Department of Ophthalmology at Rabin Medical Center in Israel (Figure 1A). The tenets of the Declaration of Helsinki were followed, the study was approved by the institutional review board, and written informed consent was obtained. At the age of 49 years, the patient experienced new-onset blurry vision, ring scotoma and photopsia. Ophthalmic evaluation at the age of 51 years revealed bilateral arterial attenuation, diffuse peripheral nasal depigmentation and inferior macular retinal pigment epithelium atrophy (Figure 2A,B). The best-corrected visual acuity was 20/20 in both eyes. The computerized Humphry visual field test (24.2 III, Sita Standard) showed bilateral upper arcuate scotoma, corresponding to the anatomic disruption in the macula (Figure 3). Fundus autofluorescence revealed a bilateral symmetric pattern of the perifoveal hyperautofluorescent ring and midperipheral nasal hypoautofluorescent specks (Figure 2C,D). Optical coherence tomography (OCT) showed preserved foveal layers with perifoveal ellipsoid zone loss (Figure 2E,F). Both scotopic and photopic responses in full-field electroretinography (ERG) were abnormally reduced in both eyes (Table 1). A multi-focal ERG 3-D representation of the waveform illustrated the presence of a foveal peak with a severely diminished perifoveal retinal response. The diagnosis was RCD.

To identify the genetic cause for RCD in Patient R2011 we performed genetic analysis. Genomic DNA was extracted from venous blood samples using a high-salt solution according to a standard protocol [30]. Genetic analysis was performed by a single molecule molecular inversion probes (smMIPs)-based panel that targets the exons and splice sites of 113 genes associated with RP and Leber congenital amaurosis, the entire *RPE65* gene and known deep-intronic variants causing pseudo-exons [31]. The only significant finding was a homozygous missense variant in the *BBS9* gene (NM_198428.3). This variant (GRCh37/hg19:Chr7:g.33192463C>T; c.263C>T) is rare, with a gnomAD allele frequency of 0.002% and no homozygotes detected. It affects a highly conserved amino acid (p.(Ser88Leu)) which is predicted to be functionally important (Figure 1C,D). The serine to leucine missense change was estimated to be pathogenic by 6/10 in silico prediction tools, while the other predictions were uncertain (Table 2). Moreover, the variant affects the last base of Exon 3, which is part of the conserved donor splice-site (Figure 1B), and was predicted to be splice-altering by 2/3 in silico prediction tools (Table 2). Unfortunately, segregation analysis was not possible due to absent intrafamilial communication.

**Figure 4:**
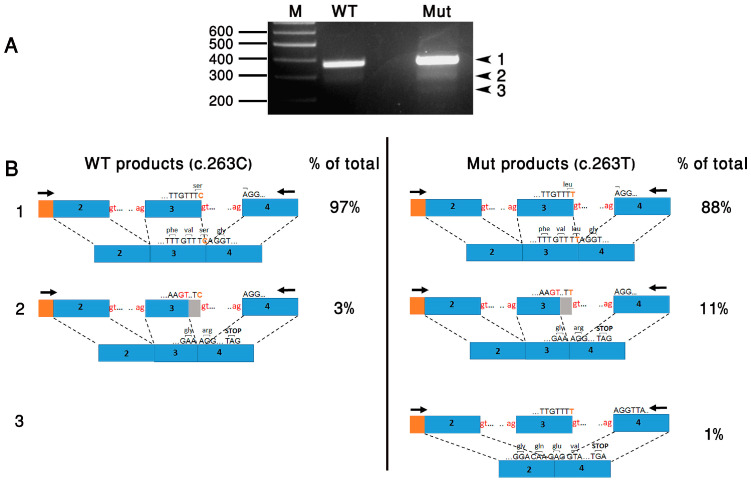
Minigene constructs and products obtained in the in vitro splicing assay. (**A**) An agarose gel image is shown, demonstrating the RT-PCR products derived from WT and mutant (Mut) constructs. M, size marker. (**B**) Shown is a schematic representation of the constructs, which include *BBS9* Exons 2, 3, and 4 (represented by blue boxes) and the introns between them (represented by straight lines). Either a C or a T is present at the last base of Exon 3. The locations of primers used for RT-PCR analysis are indicated by arrows. Vector-derived sequences are shown in orange. Also shown are the obtained products, and the relative abundance of each product in each sample. Product 1 is correctly spliced; Product 2 is an aberrantly spliced product, in which a cryptic donor site located within Exon 3 was used; Product 3 is an aberrantly spliced product in which Exon 3 was completely skipped.

To evaluate the effect of c.263C>T on splicing, we used an in vitro splicing assay approach. For this purpose, we created two minigene constructs (wild-type (WT) and mutant) harboring *BBS9* Exons 2, 3 and 4, each flanked by 150–250 bp of intronic sequences. The fragments were inserted in tandem into the pCMV-Script mammalian expression vector (Stratagene, Agilent Technologies, Santa Clara, CA, USA). Constructs were transfected into HeLa cells using the jetPEI transfection reagent (Polyplus-transfection, Illkrich, France). Cells were cultured in DMEM medium containing 10% fetal bovine serum, 1% penicillin/streptomycin and 1% glutamine (Biological Industries, Beit HaEmek, Israel) at 37 °C and 5% CO_2_. A total of 24 h following transfection, total RNA was extracted from cells with TRI reagent (Sigma-Aldrich, St Louis, MO, USA). Reverse transcription (RT) was performed with 1 μg of total RNA in a 20 μL reaction volume using 200U of M-MLV Reverse Transcriptase and 100 ng of random primers (Stratagene, Agilent Technologies, Santa Clara, CA, USA). A total of 2 μL of cDNA was subjected to PCR amplification with a forward vector-specific primer (T3) and a reverse primer located in Exon 4 (Figure 4B).

The WT construct yielded a major product of the expected size (Product 1: 364 bp) and a minor smaller product (Product 2: 346 bp). The mutant construct yielded the same two products (Products 1 and 2), as well as a third product (Product 3: 212 bp) (Figure 4A). All products were subcloned, and multiple independent clones were sequenced. This revealed that Product 1 was the normal splicing product, in which Introns 2 and 3 were correctly removed. Product 2 was an abnormal splicing product, in which a cryptic donor splice site in Exon 3 was used. This is predicted to cause a frameshift and premature translation termination (p.(Val83Argfs*11)). Product 3 was also an abnormal splicing product, in which Exon 3 was completely skipped. This is predicted to cause a frameshift and premature translation termination (p.(Asp38Glufs*34)) (Figure 4B). The quantification of band intensity (TotalLab, Gosforth, UK) revealed a difference in the relative abundance of each product between the two samples: The WT construct yielded 97% of the normal product (product 1) and only 3% of Product 2. In contrast, the mutant construct yielded 88% of Product 1, 11% of Product 2 and 1% of Product 3. In total, the aberrantly-spliced products (2 and 3) constituted 12% of the products obtained from the mutant construct, compared to 3% of the products obtained from the WT construct.

Following the identification of the *BBS9* variant as a highly probable cause for RCD in Patient R2011, we performed a thorough interview and physical examination of the patient to evaluate other systemic manifestations which may be related to BBS. The patient had normal cognitive function, with no polydactyly or other skeletal abnormalities, no truncal obesity and no history of kidney disease. In addition, she had no fertility problems (the proband has three offspring), psychiatric history, anosmia, gastrointestinal or endocrine abnormalities. The main systemic finding was epilepsy, which appeared first at the age of 35 years, and was treated by Topiramate. In addition, the patient reported severe dental problems which required several dental implants.

## 3. Discussion

IRDs are a highly heterogeneous group of disorders, with over 280 causative genes reported to date. BBS, one of the most common forms of syndromic IRD, is a genetically heterogeneous condition, which can be caused by mutations in at least 26 different genes. BBS is a pleiotropic disorder which involves multiple organs. It is inherited as an autosomal recessive condition but exhibits variable expressivity and both inter- and intrafamilial variation [32,33]. Not much is known about the underlying causes for such phenotypic variability, which may include genetic as well as environmental modifying factors. Oligogenic inheritance of BBS has been suggested in some families, in which affected individuals harbored pathogenic alleles in two or more BBS-associated genes simultaneously [34]. In one study, an oligogenic effect was suspected in 52% of the screened families, and the presence of more than two alleles in BBS-associated genes correlated in six families with a more severe phenotype and associated with specific findings, highlighting the role of the mutational load in the BBS phenotype [35]. Modifier alleles in genes not associated with BBS, such as *GLI1*, have also been reported [36]. Of note, retinal disease is the most penetrant feature in BBS, affecting up to 100% of individuals in some studies [37]. Nevertheless, the severity of eye impairment and the rate of decline of visual acuity vary greatly between and within families [33]. Moreover, at least six of the 26 BBS-associated gens have also been associated with non-syndromic RP/IRD.

The patient described here (R2011) is homozygous for the *BBS9* variant c.263C>T;p.(Ser88Leu). She was diagnosed with RCD and had no additional major features of BBS. The only systemic findings in this patient were epilepsy and dental problems. Epilepsy is found in 9.6% of BBS patients, while oral/dental abnormalities are found in 50%. Both are considered minor features [4]. Nevertheless, both epilepsy and dental problems are common in the general population, and therefore it is hard to determine whether their appearance in this patient is associated with the *BBS9* variant or coincidental.

Interestingly, the c.263C>T variant was previously reported twice in BBS patients. In both cases it was found in a heterozygous state, and in combination with two suspected pathogenic variants in another BBS-related gene (one case was homozygous for *BBS12* p.(Cys546Phe) [38]; another case was heterozygous for two *BBS1* variants: p.(Met390Arg) and p.(Arg483*) [39]). In both cases, the *BBS9* variant was not the primary causative allele, but rather considered a secondary variant contributing to the genetic burden [40]. Homozygosity for c.263C>T, as found in Patient R2011, has not been reported before. Interestingly, the minigene assay we performed demonstrated that alleles harboring either a C (WT) or a T (mutant) at the last base of Exon 3 produce a normal splicing product, but also use a cryptic donor splice-site within Exon 3 which produces an abnormal product. However, while the WT allele produces mainly the correctly-spliced transcript, the mutant allele apparently weakens the donor splice-site of Exon 3, leading to a 3.6-fold increase in the use of the cryptic splice site, as well as a small degree of exon skipping. While the relative abundance of the different splicing products, as obtained by the in vitro assay, may be different in vivo and may vary between different tissues, it demonstrates a trend. We therefore hypothesize that in individuals harboring the WT allele, the level of normal *BBS9* transcripts is sufficient to maintain normal function of the retina and other organs. In contrast, weakening of the Exon 3 donor site by the mutant allele would reduce the level of normal *BBS9* transcripts below the required threshold for normal retinal function over time and result in retinal degeneration. Therefore, based on our assay, c.263C>T is a hypomorphic allele. This is in agreement with the relatively mild phenotype observed in this patient, and the late onset of retinal degeneration. These findings indicate that the retina requires higher levels of BBS9 activity compared to other tissues. The same phenomenon has been reported for other BBS-related genes, including *BBS1* [19], *BBS2* [20], *ARL6* [21,22], *TTC8* [23,24] and *IFT172* [25]. In these genes, two severe (null) mutant alleles are usually associated with BBS. Patients with non-syndromic IRD have milder (hypomorphic) alleles. Most of these hypomorphic alleles are missense variants. One exception is a *TTC8* variant, affecting the conserved acceptor splice-site of a retina-specific exon and leading to exon skipping and in-frame deletion of 10 amino acids [24]; another one is a splice-region variant of *IFT172*, creating a novel acceptor site, which is partially used in parallel with the native acceptor site, and leads to an in-frame insertion of one amino acid [25].

The main limitation of this report is the fact that it includes a single patient. Hence, the conclusion that the observed *BBS9* variant in this patient is indeed the cause for her retinal phenotype should be taken with caution. Nevertheless, the findings described here support an expanded phenotypic spectrum associated with *BBS9* variants and indicate that variants in this gene should be considered not only in BBS patients, but also in patients presenting with phenotypes that do not meet the diagnostic criteria for BBS, including non-syndromic IRD or IRD with very mild extra-ocular manifestations.

## Figures and Tables

**Table 1:** Full-field ERG responses from Patient R2011.

		Right Eye (µV/ms)	Left Eye (µV/ms)	Normal Reference Limit (µV-Minimum/ms-Maximum)
DA 0.01	a wave (amp/implicit time)	−1.519/36.5	−9.616/45	1.0/47
	b wave (amp/implicit time)	14.3/94.5	9.754/97.5	70/108.6
DA 3.0	a wave (amp/implicit time)	−73.98/17.5	−48.26/17	−94/20.3
	b wave (amp/implicit time)	36.72/57.5	37.48/43.5	174/56.8
DA 10.0	a wave (amp/implicit time)	−91.94/15.5	−69.22/15.5	−113/15.42
	b wave (amp/implicit time)	51.5/53.5	45.83/35	200/54.3
LA 3.0	a wave (amp/implicit time)	−10.63/12.5	−7/16	−15.2/16.7
	b wave (amp/implicit time)	30/32.5	40.52/32.5	56.6/34
LA FLICKER 30 Hz	Amplitude/ time (msec)	32.21/30.5	18.31/29	57.2/30.3
	Trough (msec)	15	15	15.3

DA, dark-adapted; LA, light-adapted; amp, amplitude.

**Table 2:** In silico predictions for the *BBS9* c.263C>T variant described in this study.

Prediction Tool	Prediction (Score)	Scale
Mutation Taster	Deleterious (1)	0–1, higher scores reflecting a greater likelihood that the variant is deleterious
Missense prediction tools		
Revel	Deleterious (0.76)	0–1, higher scores reflecting a greater likelihood that the variant is deleterious
Polyphen2	Deleterious (1)	0–1, higher scores reflecting a greater likelihood that the variant is deleterious
DANN	Deleterious (1)	0–1, higher scores reflecting a greater likelihood that the variant is deleterious
MetaLR	Deleterious (0.71)	0–1, higher scores reflecting a greater likelihood that the variant is deleterious
BayesDel	Deleterious (0.13)	(−1.29334)–0.75731, Deleterious: >(−0.0570105)
SIFT	Uncertain (0.001)	0–1, Deleterious: <0.001
FATHMM	Uncertain (−1.74)	(−16.13)–10.64, Deleterious: <(−4.14)
PrimateAI	Uncertain (0.74)	0–1, Deleterious: >0.803
AlphaMissense	Uncertain (0.477)	0–1, Deleterious: >0.761
Splice-site prediction tools		
dbscSNV Ada	Deleterious (0.99)	0–1, higher scores reflecting a greater likelihood that the variant is deleterious
dbscSNV RF	Deleterious (0.74)	0–1, higher scores reflecting a greater likelihood that the variant is deleterious
SpliceAI	Uncertain (0.12)	0–1, Deleterious: >0.2

## Data Availability

The *BBS9* variant identified in this study was submitted to the Leiden Open Variation Database (LOVD) (http://www.lovd.nl, accessed on 13 March 2024).
